# Quantification of serotonin O-sulphate by LC-MS method in plasma of healthy volunteers

**DOI:** 10.3389/fphar.2014.00062

**Published:** 2014-04-08

**Authors:** Raimonds Lozda, Indulis Purviņš

**Affiliations:** Pharmacology Group, Department of Internal Diseases, Riga Stradin's UniversityRiga, Latvia

**Keywords:** serotonin-O-sulfate, serotonin, hydroxytriptophane, biomarker, depression

## Abstract

The objective of this study was to test the hypothesis that serotonin O-sulphate (5-HT-SO4) could be quantified in human plasma using modern liquid chromatography–mass spectrometry (LC-MS) method as well as develop and validate that method. First, a suitable LC-MS method for detection of 5-HT-SO4 in human plasma samples was developed and validated. Second, a Pilot phase involving four healthy volunteers was executed, where a basal plasma level of 5-HT-SO4 was measured for all subjects and for one after the intake of 100 mg of a 5-hydroxytryptophan (5-HTP) -containing food supplement used to promote serotonergic stimulation of the central nervous system. The basal level of 0.9–2.8 ng/mL of 5-HT-SO4 was observed. The changes of plasma 5HT-O-SO4 showed 1.2 ng/mL before and 22.6 ng/mL 1 h after stimulation. Finally, nine healthy volunteers were selected for the Study phase, where a basal plasma level of 5-HT-SO4 was measured before and after the intake of 5-HTP. One hour after stimulation, six study subjects showed a decrease in 5-HT-SO4 levels while three subjects showed an increase. The changes of plasma 5HT-O-SO4 from the Study phase showed an average 5-HT-SO4 level of 19.2 ng/mL before and 15.7 ng/mL 1 h after stimulation indicating ability of method to emphasize quantitative changes. This was the first study in which naturally occurring 5-HT-SO4 was detected in the samples of human plasma obtained from healthy volunteers. The method developed herein is specific to the measurement of 5-HT-SO4, sensitive enough to quantify intra-individual changes in the samples of plasma and opens up new possibilities to evaluate pathways of serotonin metabolism by minimally invasive methods. The discovery of novel biomarkers using such approaches is increasingly required to expedite development of mechanism-based therapeutics and patient stratification.

## Introduction

Major depressive disorder (MDD) affects 4–6% of adolescents, which, when left untreated, subsequently leads to an immediate high suicide risk, long-term chronicity and a poor psychosocial outcome (Masi et al., 2010). Though effective treatments for mood and anxiety disorders have been available for more than 40 years, 30–50% of depressed patients and 25% of patients with anxiety disorder do not respond sufficiently to first-line treatment with antidepressants. Because patients with MDD may not respond to antidepressants for several weeks or longer, a biomarker that predicts treatment effectiveness after only 1 week could be clinically useful. Additionally, the discovery of novel biomarkers using minimally invasive approaches is increasingly required to expedite drug development in the era of mechanism-based therapeutics and patient stratification (Masi et al., 2010).

The monoamine deficiency theory posits that the underlying pathophysiological basis of depression is a depletion of the neurotransmitters serotonin (5-HT), norepinephrine (NE) or dopamine (DA) in the central nervous system (CNS) (Hasler, 2010). The well-known marker of serotonin metabolism in the brain is 5-hydroxyindoleacetic acid (5-HIAA) in cerebrospinal fluid (CSF), because a correlation between low levels of 5-HIAA in the CSF and suicidal behavior has been reported (Asellus et al., 2010). Additionally, the effectiveness of antidepressant treatments has been evaluated based on the CSF 5-HIAA approach. However, the use of lumbar puncture is restricted for medical and ethical reasons. On the other hand, increased plasma levels of 5-HIAA and 5-HT have been observed in depressed patients and that plasma 5-HIAA directly correlates with the severity of depression (Mitani et al., 2006). Thus, the laboratory value of 5-HIAA as a serotonin metabolism biomarker is defined by the ability to measure this compound both in CSF and plasma. Nevertheless, the clinical significance of 5-HIAA in CSF is greater than that in plasma. From the practical convenience point of view, a biomarker emphasizing CNS specific 5-HT metabolism that does not require a spinal puncture would be the most valuable. For such an important role, the serotonin catabolite 5-HT-SO4, could be evaluated.

A sulphation reaction of serotonin to its biotransformation product 5-HT-SO4 was described in the middle of the last century (Kishimoto et al., 1961). Animal experiments during later years revealed that 5-HT-SO4 is the final product of serotonin catabolism, which is rapidly excreted from the organism (Hidaka et al., 1969; Rose and Bleszynski, 1971). The same compound was also found in human CSF (Tyce et al., 1985). During the 1980 and 1990s, 5-HT-O-SO4 was intensively investigated. In one study, considerable amounts of acid-hydrolysable conjugates of DA, NE, and 5-HT were detected in the CSF of normal individuals. The amounts of conjugated amines were small in comparison to the amounts of homovanillic acid and 5-HIAA (Tyce et al., 1986). In the other study performed with CSF from humans and ventriculocisternal perfusion of African green monkeys, sulphates of NE, DA, and 5-HT were also found to be present in the CSF of laboratory animals and humans. Furthermore, the amount of sulphated amines in human CSF always greatly exceeds the amount of free amines. The ratio of 5-HT-O-SO4 in perfusates to 5-HT-O-SO4 in plasma increased after intravenous (i/v) injection of 5-HT-O-SO4 (300–400 μ g/kg). The ratio of amine sulphate in the perfusate to amine sulphate in plasma was greater for 5-HT-O-SO4 than for DA-O-sulphate at 60 and 100 min after i/v injection. Finally, because 5-HT-O-SO4 could not be detected in the plasma of monkeys or humans under normal conditions, it was confirmed that the 5-HT-O-SO4 in ventriculocisternal perfusates undoubtedly originated in the central nervous system (Tyce et al., 1985).Failure to detect this compound in plasma during the trials described above is likely due to the early development stage of high-performance liquid chromatography (HPLC) methods.

In recent years, several studies have been performed on marine molluscs to determine 5-HT-O-SO4 levels in their nervous systems. Indeed, 5-HT-O-SO4 was detected in the somato of the serotonergic metacerebral cells of Aplysia and Pleurobranchaea as well as the pedal G cells of Pleurobranchaea. The most intriguing finding, however, was that the fate of 5-HT in the central nervous system depends upon its release location (Stuart et al., 2003, 2004). Unfortunately, the clinical significance of 5-HT-O-SO4 has thus far been lessened, likely due to its absence in the peripheral blood circulation and the better-established 5-HIAA method.

As another potential source of 5-HT-O-SO4 a urine was investigated. Laboratory test animals have been shown to excrete appreciable amounts of serotonin-O-sulfate after the administration of large doses of serotonin. However, the presence of this serotonin metabolite in the urine of normal man given oral loads of serotonin was not detected, but in the urine of patients with carcinoid tumors it has been quantified by utilizing ion exchange resins (Davis et al., 1966). So, the findings with urine showed lack of 5-HT-O-SO4 under physiological circumstances, probably due to insensitiveness of the methods employed. However, since there is a lack of data related to circadian rhythm of 5-HT-O-SO4 we decided to concentrate on another body fluid-plasma as more potent source for the future investigation.

The latest scientific data have allowed us to hypothesize that the measurement of 5-HT-O-SO4 in human plasma by modern LC-MS methods is sensitive enough to detect small amounts of the compound in human plasma. The aim of our research was thus to develop an appropriate chromatographic method based on a minimally invasive approach to measure 5-HT-O-SO4 in human plasma and to test that method in clinical practice on healthy volunteers.

## Materials and methods

This study was approved by the independent ethics committee for clinical research of medicines and pharmaceutical products in Latvia namely “Neatkarīgā zāļu un farmaceitisko produktu klīniskās izpētes ētikas komiteja” as well as the board of Latvian Institute of Organic Synthesis. All human subjects provided informed consent to participate, and written informed consent was obtained from all participants.

### Subjects

Thirteen healthy volunteers were enrolled in the trial. The characteristics of the subjects are summarized in Table 1.

**Table 1:** **Study subjects characteristics**.

**Study ID**	**Age**	**Gender**	**Remarks**
**PILOT PHASE**			
SF 1	42	M	
SF0-1	34	F	Control group not receiving 5-hydroxytryptophan
SF0-2	36	F	Control group not receiving 5-hydroxytryptophan
SF0-3	27	F	Control group not receiving 5-hydroxytryptophan
**STUDY PHASE**			
SF-2	64	M	
SF-3	54	M	
SF-4	43	M	
SF-5	29	M	
SF-6	20	F	
SF-7	42	F	
SF-8	28	F	
SF-9	46	F	
SF-10	57	F	

The age is given in years, abbreviation: M, refers to male; F, female. In the Pilot phase the subject SF 1 and all Study phase subjects underwent the serotonergic stimulation.

The eligible age range for subjects in the study was 18–80 years old. Both genders were also eligible for the study. Participation in this study was also determined by the following inclusion criteria: subjects in general good health and in whom the use of any of the study food supplement compounds were not contraindicated; subjects who could communicate with the study personnel and complied with study requirements; subjects not suffering from depressive mood disorders; subjects not taking any medication that may increase serotonin levels in the organism; and female subjects that had not been pregnant or breast-feeding. The following criteria led to exclusion from the study: subjects with abnormal screening laboratory results that were considered clinically significant by the investigator; subjects with diagnosed depressive mood disorders, history of antidepressant treatment or severe renal insufficiency; subjects who had participated in a clinical trial in the previous 30 days; subjects with known allergies to any of the active ingredients or excipients of the study compound; and subjects not complying with all of the inclusion criteria. Four volunteers were involved in the Pilot and 9 in the Study phase.

### Study protocol

This study was designed to quantify intra-individual changes of plasma 5HT-O-SO4 using a cohort of healthy subjects.

#### Pilot phase

Under fasting conditions, baseline blood samples (0 h) from the four study subjects were collected in 20 mL vacuum tubes. To define the sensitivity of the method to quantitatively detect intra-individual changes of the 5-HT-O-SO4 levels, serotonergic stimulation of one of the study subjects was performed. The subject ingested two capsules of a food supplement containing 100 mg of 5-HTP, 1 h after which a second blood sample was collected. All samples were subsequently centrifuged, plasma removed and placed into a 12 × 75 polypropylene microcentrifuge tubes, thereafter frozen to a minimum temperature of −24**°**C. Ten days later, detection of 5-HT-O-SO4 was performed using LC-MS methods.

#### Study phase

As in the Pilot study, baseline blood samples (0 h) of all study subjects were collected in 20 mL vacuum tubes. In this study, all of the subjects underwent the serotonergic stimulation process to ascertain the sensitivity of the method to quantitatively detect intra-individual changes of the 5-HT-O-SO4 levels. One hour after ingesting two capsules of a food supplement containing 100 mg 5-HTP, a second post-stimulation blood sample was obtained from each subject. All blood samples until analysis were handled as per Pilot phase. HPLC detection of 5-HT-O-SO4 was performed on the samples after 20 days. All of the blood samples were collected according to good clinical practice at a certified medical institution.

### Food supplement used in the study

To stimulate the serotonergic system, we choose to use an orally administered food supplement containing the serotonin precursor 5-HTP.

The 330.5 mg capsules containing 50.5 mg 5-HTP (as Griffonia simplicifolia seed extract supplied by Synpharma International Ltd, UK), 1.25 mg pyridoxine hydrochloride and 200 mg magnesium oxide are legally available on the EU market. The rationale for using a product with this composition is described as follows. Pyridoxine (vitamin B6) is a cofactor for l-amino acid decarboxylase, an enzyme that catalyses the decarboxylation of a variety of aromatic amino acids—it converts 5-HTP to serotonin (Turner et al., 2006). The magnesium oxide serves as a substitution for magnesium depletion to mimic the fact that dietary magnesium intake often tends to be lower than recommended (Marier, 1986) and may play a potential role in depressive disorders (Szewczyk et al., 2008). The intention of using a combination supplement was to exclude possible effects of dietary deficiencies leading to impaired serotonin metabolism. The rationale for 5-HTP dosages and administration regimen was based on findings that the lowest average dose used in clinical trials was 100 mg, and its maximal plasmatic concentration was achieved within 1–2 h after administration (Turner et al., 2006).

We avoided the use of pure 5-HTP because nausea is a general side effect often encountered during its administration (Jacobs et al., 2010). We also decided against the addition of carbidopa, which is known to prevent peripheral conversion of 5-HTP to 5-HT. As previously reported, the average systemic availability of oral 5-HTP was approximately 70% (Turner et al., 2006), and there is no consensus as to whether the addition of carbidopa increases the efficacy of 5-HTP (Zmilacher et al., 1988).

### Materials and reagents

The 5-HT-O-SO4 was purchased from Chemos GmbH. All solutions were prepared or purchased as follows: acetonitrile (Merck, LiChrosolv), formic acid (Fluka), deionized water (*R* > 18 MΩ/cm, TOC < 10 ppb) produced by a Millipore-Q water system (Bedford, MA, USA).

### Instrumentation

Analyses were performed on a liquid chromatography (Acquity) - mass spectrometer (Waters Quattro Micro) tandem device using MassLynx 4.1 software for data registration.

A Hydrophilic Interaction Liquid Chromatography (HILIC) type sorbent on a gradient regime achieved chromatographic separation of the sample components.

Samples for analyses were prepared by precipitation of plasma proteins with acetonitrile followed by purification with a solid phase extraction method using the HybridSPE solid phase precipitation cartridge.

#### Chromatographic and mass spectrometer conditions

The analytical column was a Waters Acquity BEH HILIC (1.7 μm, 2.1 × 100 mm), with a 5 μ l injection volume. The mobile phase consisted of acetonitrile (phase A) and a 0.1% formic acid aqueous solution (phase B). The following mobile phase parameters were used for these measurements: gradient—0 min; 85% A, 2.5 min; 55% A, 3 min; 55% A, 5 min 85% A, 4 min; and flow—0.2 mL/min.

The mass spectrometer was a Micromass Quattro Micro using a triple quadrupole mass spectrometer. The metabolite 5-HT-O-SO4 was ionized in a positive mode. The tandem mass spectrometry (MS/MS) parameters are described in Table 2.

**Table 2:** **MS/MS parameters of serotonin O-sulfate**.

**Compound**	**MRM transition**	**Cone voltage, V**	**Collision energy, eV**
Serotonin	257 >> 160	20	19
sulfate	240 >> 160	35	17

MRM refers to multiple reaction monitoring.

### Preparation of calibration standard solutions and samples for analysis

Calibration standards for human studies were prepared by dilution of a serotonin O-sulphate stock solution in 80% methanol (*C* = 93 μg/mL) to obtain 6 calibration samples with concentrations ranging from 10 to 225 ng/mL.

For human studies, serotonin O-sulphate was quantified by the standard addition method. Three aliquots (100 μ L) of each plasma sample were spiked with 20 μ L of serotonin O-sulphate standard solution of various known concentrations (2 samples) or 20 μ L of water. Next, 300 μ L of 1% formic acid solution in acetonitrile was added to each plasma sample and mixed. Samples were centrifuged (10 min at 10,000 rpm), and the resulting supernatant was loaded onto the HybridSPE cartridge. The eluate was then collected in an HPLC vial, and a 5 μ L aliquot was injected into the LC-MS system for analysis.

### Statistics

Statistical analysis of the study changes of plasma 5HT-O-SO4 was performed using MS Excel 07. The changes of plasma 5HT-O-SO4 from human studies were analyzed using the 5-HT-O-SO4 measurements taken at both baseline (0 h) and post-stimulation (1 h) points. Data were analyzed through a paired *t*-test.

## Results

### HPLC method development

The method for detection of 5-HT-O-SO4 in the human plasma was validated according to “Draft guideline on validation of bioanalytical methods” published by the European Medicines Agency (EMEA, 2009) in terms of specificity, linearity, recovery, accuracy, and precision.

#### Specificity of the method

**Figure 1:**
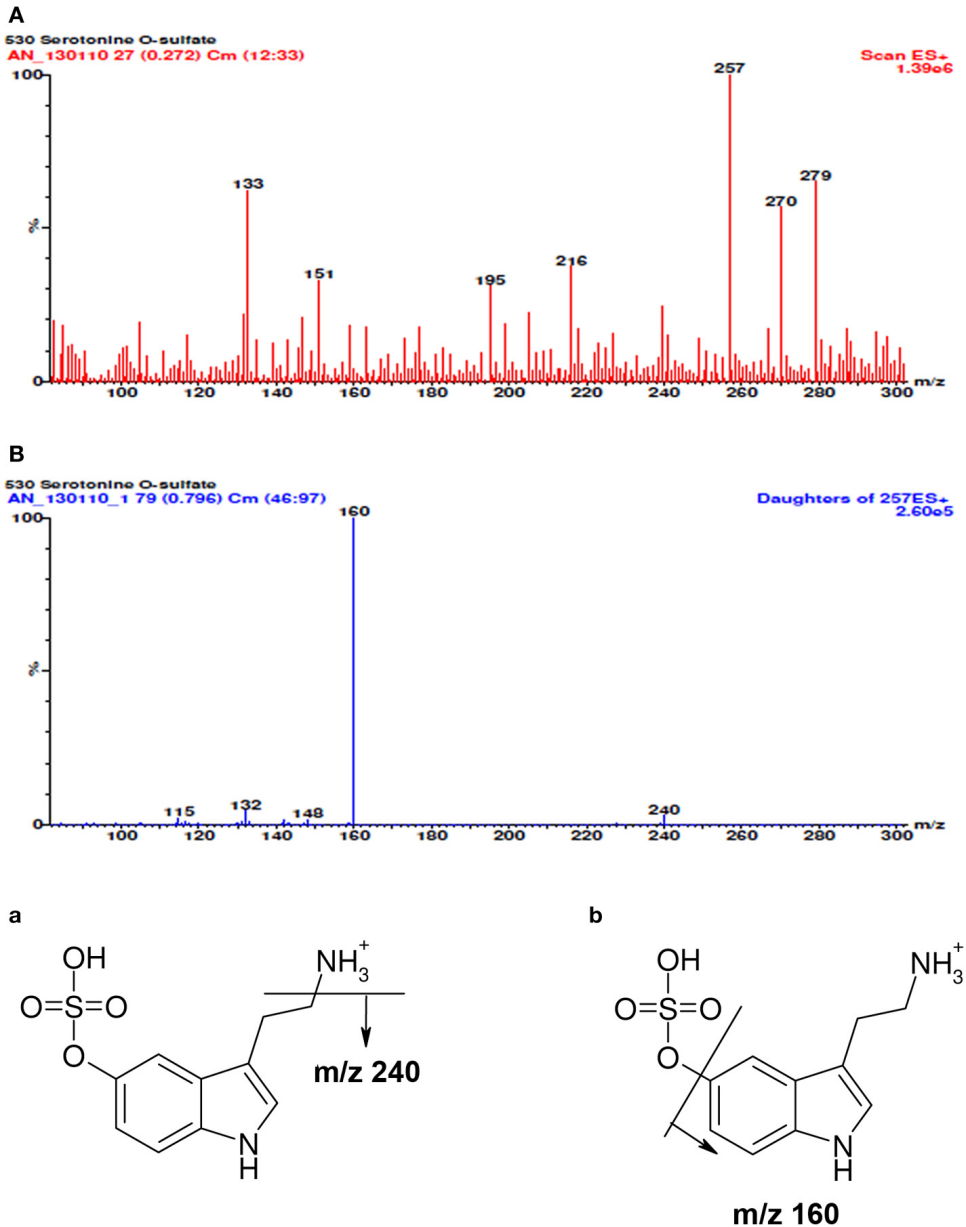
**The full mass spectra of 5-HT-SO4, daughter ions and possible scheme of fragmentation**. In the spectra **(A)** besides molecular ion [M+H]^+^ with m/z value of 257 a sodium adduct [M+Na]^+^ (m/z 279) and some median intensity *cleavage products* (m/z- 240; 218; 195) are seen. The mass spectra **(B)** of molecular ion decay is linked to the elimination of ammonium (m/z 240) and sulphuric acid (m/z 160 the most intensive cleavage ion). Fragmentation scheme of 5-HT-SO4 is seen at the end of the picture. The **(a)** shows cleavage of ammonia **(b)** sulfuric acid residue and the corresponding m/z values m/z.

Tandem mass spectrometric analysis (MS/MS) was made in a positive-ion mode (ESI +). The total ion current full mass range of 5-HT-SO4 and daughter ions is shown on Figure 1. The electrospray ionization of 5-HT-SO4 was weak. Thus, for the further quantitative analysis a following ion transition was used: (257 >> 160) + (240 >> 160).

**Figure 2:**
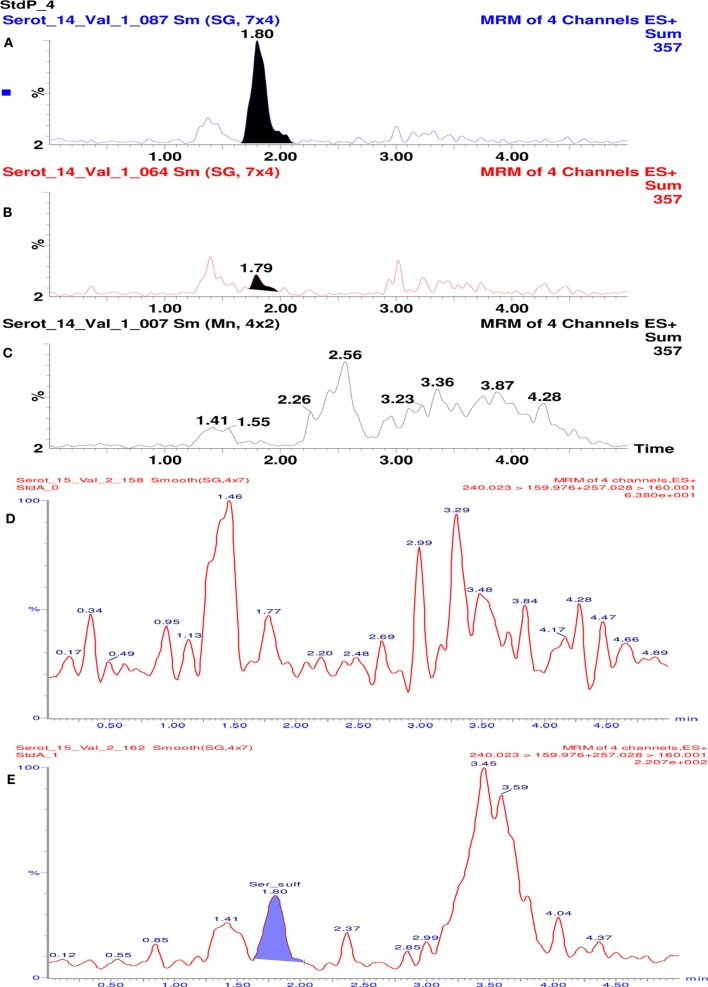
**Chromatograms of 5-HT-SO4 samples and MRM chromatograms**. The **(A)** shows plasma standard solution (containing 96 ng/mL of 5-HT-SO4) where 1.80 min retention time peak is seen; **(B) – in** the “pure” plasma 1.8 min retention time peak is seen; **(C)** – purified water where no 1.79–1.80 min peak is observed. **(D)** shows MRM chromatogram of analytical solution made of 5% serum albumin. The signal with a retention time ~1.8 min. is not observed. **(E)** shows 5% serum albumin solution containing 5-HT-SO4 (10 ng/mL). The signal with retention time 1.80 min, which corresponds to the site analyzed is observed.

Specificity of the method was assessed visually by comparing multiple reaction monitoring (MRM) chromatograms of plasma sample spiked with serotonin O-sulfate, samples of plasma and purified water. As seen in Figure 2, in the plasma based calibration standard **(A)** and plasma **(B)** some 1.79–1.80 min retention time peaks can be observed. The purified water samples treated similarly do not show such signal **(C)**. This signal might be induced by native content of serotonin sulphate found in plasma samples. Conclusion was reached because in the analytical solution made of 5% serum albumin such signal was not seen (Figure 2).

For the MRM chromatograms shown on Figure 2D the test solution of 5% serum albumin (buffered to pH = 7 in a phosphate buffer) was prepared.

Further the 5% serum albumin (buffered as above) was dissolved into 0.9% NaCl solution and 10 ng/mL of 5-HT-SO4 added. The MRM chromatogram is shown on Figure 2E.

The results obtained lead to conclusion that the method developed is specific to the compound of interest—5-HT-SO4.

#### The linearity and working range

The linearity of detection was evaluated three times in different days by analyzing calibration standard solutions of 5-HT-SO4.

**Figure 3:**
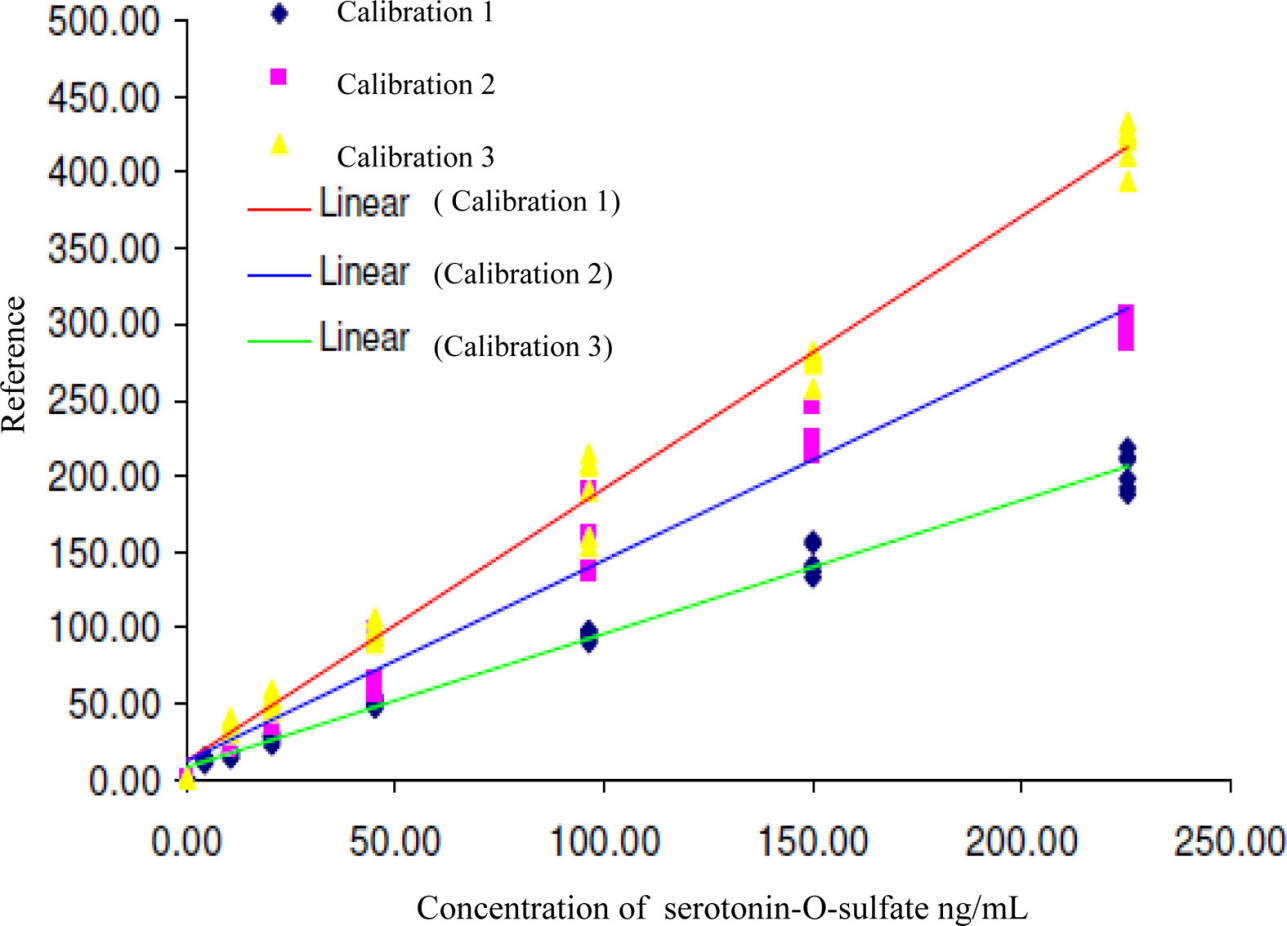
**Calibration lines of 5-HT-SO4 during three different days of analysis**. The concentration of 5-HT-SO4 in the plasma samples is given prior to treatment. For the Calibration 1 concentration range was 4.4–225.2 ng/mL, for the Calibration 2 and 3 the range was 10.7–225.2 ng/mL.

In the first analysis, the concentration range was 4.4 to 225.2 ng/mL, 2nd and 3rd analysis,—10.7 to 225.2 ng/mL. The calibration lines are shown on the Figure 3.

Statistical parameters of the calibration lines are described in Table 3.

**Table 3:** **The statistical parameters of 5-HT-SO4 calibration lines**.

**Parameter**	**Value**		
	**1. Calibration**	**2. Calibration**	**3. Calibration**
Concentration range, ng/mL	4.4–225.2	10.7–225.2	10.7–225.2
Correlation coefficient squared, R^2^	1.0	1.0	1.0
Slope, A	0.9	1.3	1.8
Free member, B	7.5	11.8	11.3
Range of free member (α = 0.05)	5.1–10.4	5.1–19.6	6.0–17.7
Statistical significance of free member	YES	YES	YES

The “1. Calibration” refers to the range of 4.4–225.2 ng/mL, “2. Calibration” and “3. Calibration” to the range of 10.7–225.2 ng/mL performed during three different days of analysis. A free member of calibration lines statistically differs from zero in all the cases.

This method resulted in a linear relationship between concentration of the analyte (10–225 ng/mL) and mass spectral signal of 5-HT-SO4 with a calibration curve correlation coefficient of >0.98.

The data obtained matches to finding that the peak of 5-HT-SO4 in the plasma samples was observed. The preparation of calibration standard solutions for the further human studies were done using concentration range 10 to 225 ng/mL.

**Figure 4:**
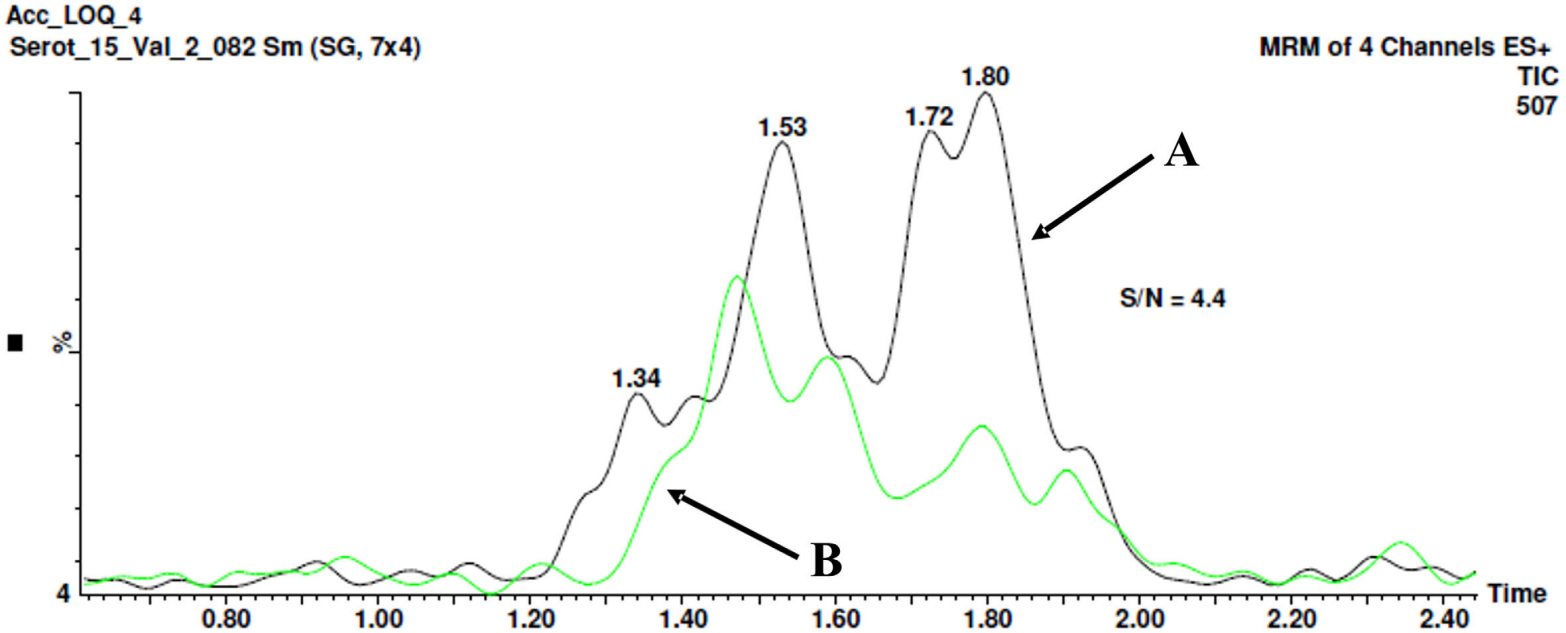
**The method for signal to noise (S/N) ratio determination**. Chromatogram B shows the “clean” plasma sample no spiking with 5-HT-SO4. The peak with a retention time 1.8 min. is seen, area- 12.3. Chromatogram A shows the plasma sample spiked with 26.5 ng/mL of 5-HT-SO4, the peak 1.8 min, area- 54.4. The resultant S/N ratio is 4.4.

The optimal detection limit of 5-HT-SO4 in the plasma sample was determined to be 26.5 ng/mL. The signal to noise (S/N) ratio was determined to be 4.4 as shown on Figure 4.

The both “clean” plasma sample not spiked and the sample spiked with 26.5 ng/mL of 5-HT-SO4 were used.

#### Recovery

Four different concentrations of 5-HT-SO4 were used for a recovery testing by adding them to the pure plasma samples. Six samples for each concentration level were prepared. The results obtained are described in Table 4.

**Table 4:** **The results of 5-HT-SO4 recovery testing**.

**Serotonin O-sulfate, ng/mL**	**Recovery ± *SD*, %**
26.8	118.2 ± 5.3
45.5	120.7 ± 7.9
59.9	104.2 ± 4.5
156.0	120.2 ± 6.6
The average recovery, %	115.8
Relative *SD*, %	6.8

The accuracy of results is little dependent on the amount of serotonin O-sulfate added.

The HPLC method gave correct 5-HT-SO4 detection results, which were justified by the average level of recovery of the analyte at 116 ± 8%.

The relatively high interval of recovery can be explained due to the matrix effect, caused by somewhat dirty plasma samples significantly affecting the analytical signal of the compounds.

#### Intralaboratory reduplication

Accuracy of the method was tested by the preparation of plasma samples during three different days, in six parallel samples. The results described in Table 5.

**Table 5:** **Intralaboratory precision and reduplication of the method**.

**Day**	**Medium reduplication ± *SD*, %**	**Relative *SD* for parallel samples %**	**Accuracy, %**
Day 1	114.7 ± 8.2	12.0	12
Day 2	104.2 ± 4.5	4.3	
Day 3	94.5 ± 5.7	10.1	

Each plasma sample contains 59.9 ng/mL of 5-HT-SO4.

Intra-laboratory accuracy of the method over a 3-day period was characterized by a standard deviation of ±12%.

Taking into account above mentioned results the method was concluded to be a suitable technique for measuring basal 5-HT-SO4 levels in human blood samples as well as quantitative changes. Considering this, we decided to test this method in a Pilot study involving healthy volunteers.

### Pilot study

The purpose of the Pilot study was to apply the method developed in the first-in-humans study. The concentrations of basal plasma 5-HT-SO4 for all four subjects were measured. The subject SF1 ingested two capsules of a food supplement containing 100 mg 5-HTP. To test the intra-individual sensitivity of the method, a second blood sample from one subject (SF1) was obtained 1 h after serotonergic stimulation.

Each plasma sample was analyzed three times—without 5-HT-SO4 and by addition of 5-HT-SO4 standard in two concentration levels (28 and 54 ng/mL). The average results for each sample are described in Table 6.

**Table 6:** **Concentrations of plasma 5-HT-SO4 obtained from the Pilot study subjects**.

**Sample**	**Concentration of serotonin-O-sulfate**		
	**Average ng/mL**	***SD* ng/mL**	**RSD %**
SF 1 (Start)	1.2	±0.04	3.0
SF 1 1 h after 5-HTP ingestion	22.6	±0.03	0.2
SF0-2 control	1.9	±0.11	5.9
SF0-3 control	0.9	±0.09	9.8
SF0-4 control	2.8	±0.31	11.1

The calculation of average ng/mL and relative standard deviation (RSD) is based on 3 analyses—no 5-HT-SO4 added, 28 and 54 ng/mL of 5-HT-SO4 added.

### Study phase

Our main interest was to ascertain quantitative differences of basal 5-HT-SO4 levels and intra-individual sensitivity of the quantitation obtained in the Pilot study on a larger number of subjects. Thus, after measurement of the basal 5-HT-SO4 levels, all subjects were exposed to serotonergic stimulation and a second blood sample was analyzed.

Each plasma sample was analyzed three times as per Pilot study.

The concentrations of plasma 5-HT-SO4 obtained from the study subjects are described in Table 7.

**Table 7:** **Concentrations of plasma 5-HT-SO4 obtained from the study subjects and data of statistical analysis**.

**Sample**	**Concentration of serotonin-O-sulfate at baseline**			**Concentration of serotonin-O-sulfate 1 h after 5-HTP ingestion**			**Relative change of concentration**
	**Average, ng/mL**	***SD*, ng/mL**	**RSD %**	**Average, ng/mL**	***SD*, ng/mL**	**RSD %**	**ng/mL**
SF-2	**20.6**	1.2	5.0	**14.9**	0.5	3.5	**5.6**
SF-3	**22.7**	0.6	2.7	**16.4**	0.6	3.7	**6.4**
SF-4	**23.6**	2.4	10.1	**14.9**	0.2	1.4	**8.6**
SF-5	**17.0**	0.1	0.6	**10.1**	0.4	4.3	**6.8**
SF-6	**28.1**	1.1	3.8	**21.9**	1.0	4.3	**6.2**
SF-7	**26.1**	1.2	4.7	**27.3**	0.6	2.1	**−1.1**
SF-8	**11.6**	0.3	2.9	**12.6**	0.3	2.3	**−1**
SF-9	**15.0**	2.3	15.3	**17.6**	0.3	1.5	**−2.6**
SF-10	**8.1**	0.1	1.1	**5.3**	0.01	0.2	**2.8**
Average in the group	**19.2**	6.8	35.3	**15.7**	6.4	40.8	
***T*−TEST: PAIRED TWO SAMPLE FOR MEANS**							
*P*(*T* ≤ *t*) one-tail	0.02	t Critical one-tail	1.9	*P*(*T* ≤ *t*) two-tail	0.03	t Critical two-tail	2.3

The calculation of average ng/mL and RSD is based on 3 analyses—no 5-HT-SO4 added, 28 and 54 ng/mL of 5-HT-SO4 added.

In six study subjects, a decrease in 5-HT-SO4 levels was observed 1 h after 5-HTP ingestion.

Three subjects, however, showed an increase of 5-HT-SO4 1 h after 5-HTP ingestion.

Paired Two Sample for Means analysis showed statistically significant differences between individual measurements.

In summary, we developed a suitable LC-MS method for the detection of 5-HT-SO4 in human plasma samples based on a minimally invasive laboratory method.

During the Pilot study, we detected 5-HT-SO4 in the plasma samples of healthy volunteers for the first time. The Study phase confirmed the suitability of the method developed for clinical application by detecting basal 5-HT-SO4 levels in plasma samples and its ability to emphasize quantitative changes.

## Discussion

The significance of our research is that, contrary to earlier findings, we have measured evidence of naturally occurring 5-HT-SO4 in human plasma. The present finding opens up new possibilities for monitoring minor 5-HT metabolism pathways in the peripheral blood stream. Furthermore, 5-HT-SO4 could potentially be employed as a biomarker of MDD severity and antidepressant treatment efficacy (Mitani et al., 2006), similarly to 5-HIAA.

The key limitations of this method in the past include the lack of knowledge on CNS-specific site of 5-HT-SO4 appearances and the fact that monitoring of this compound was only possible in the CSF. Thus, 5-HIAA had many more advantages from a feasibility and convenience standpoint. However, we now have evidence that serotonin O-sulphate is present in the same body fluids as other 5-HT metabolites. Moreover, taking into account conclusions regarding doubtful CNS origin of 5-HT-SO4 (Tyce et al., 1985), we have a potential tool to monitor central serotonergic metabolism in the peripheral blood stream.

For the justification for the HPLC method we employed and its comparison to other available a following concern was taken into account. Neurotransmitters can be analyzed by gas chromatography (GC) but a derivatization step is necessary. Therefore, LC or capillary electrophoresis (CE) has been more frequently used, applying electrochemical fluorescence or UV detection. These methods often require a derivatization of the analytes or do not provide enough sensitivity or specificity. Therefore, other detection modes, such as mass spectrometry, have been used. MS has recently made a big impact on the determination of this type of compounds and provides several advantages over conventional methods, because structural information can be given, and moreover, better sensitivity and selectivity can be achieved (González et al., 2011).

Despite other studies related to 5-HT-SO4 research used to employ CE with laser-induced fluorescence (LIF) technique we concerned the statement that CE-LIF could not fulfill the expectation of becoming a routinely applied technique in clinical laboratories or pharmaceutical industry. The limited number of real applications compared to HPLC methods justifies this. The reasons are the relatively high cost of instrumentation, the lack of ready to use applications and the need for careful design of method development requiring skilled and experienced analyst (Szöko and Tábi, 2010).

However, several questions raised by our findings need to be addressed. The first is whether the 5-HT-SO4 found in plasma indeed has CNS origin. The second is to provide a possible explanation of the elevated or lowered sulphate levels observed in the present research. Future investigations are needed to explain these findings.

### Conflict of interest statement

The authors declare that the research was conducted in the absence of any commercial or financial relationships that could be construed as a potential conflict of interest.
